# The voice of nurses as a means to promote job engagement*

**DOI:** 10.1590/1518-8345.3193.3208

**Published:** 2019-10-28

**Authors:** Isabel Sanclemente-Vinue, Carmen Elboj-Saso, Tatiana Iñiguez-Berrozpe

**Affiliations:** 1 University of Zaragoza, Department of Psychology and Sociology, Zaragoza, Zaragoza, Spain.

**Keywords:** Nurses, Occupational Health, Qualitative Analysis, Health Promotion, Discussion Forums, Psychological Burnout, Enfermeiras e Enfermeiros, Saúde do Trabalhador, Análise Qualitativa, Promoção da Saúde, Fóruns de Discussão, Esgotamento Psicológico, Enfermeros, Salud Laboral, Análisis Cualitativo, Promoción de la Salud, Foros de Discusión, Agotamiento Psicológico

## Abstract

**Objective:**

The objective of this study was to establish the factors that induce nursing professionals to present engagement with their work environment.

**Method:**

Qualitative study, using the communicative methodology, with nursing professionals from public and private centers in the city of Huesca. The statements were collected through communicative stories and discussion groups.

**Result:**

The methodology used has allowed the establishment of a set of engagement promotion measures in the studied environment. These measures are framed in the three main categories analyzed: the systemic or structural variables, the subject-oriented variables, and those that refer to the relationships between the subjects.

**Conclusion:**

Knowledge of the situation regarding engagement among professionals, and the issues that encourage or hinder its appearance, is essential in establishing measures that contribute to its development. The use of qualitative techniques has allowed for the discovery of situations that would have gone unnoticed. After analyzing the interviews and the discussion groups, the following were present: an important lack of recognition that the participants experience, and that contribute, in their opinion, to the appearance of burnout syndrome, and, the lack of cohesion as a collective.

## Abstract (pt)

**Objetivo:**

O objetivo deste estudo foi estabelecer os fatores que induzem os profissionais de enfermagem a apresentar *engagement* em seu ambiente de trabalho.

**Método:**

Estudo qualitativo, utilizando a abordagem comunicativa, conduzido com profissionais de enfermagem de centros públicos e privados da cidade de Huesca. Os discursos foram coletados por meio de relatos comunicativos e grupos de discussão comunicativa.

**Resultados:**

A metodologia utilizada permitiu o estabelecim ento de um conjunto de medidas de promoção do *engagement* no ambiente estudado. Essas medidas são enquadradas nas três categorias principais analisadas: as variáveis ​​sistêmicas ou estruturais, as variáveis ​​que se referem aos sujeitos e as que se referem às relações entre os sujeitos.

**Conclusão:**

Conhecer a situação de *engagement* dos profissionais e as questões que estimulam ou dificultam sua manifestação são essenciais para estabelecer medidas que contribuam para o seu desenvolvimento. O uso de técnicas qualitativas nos permitiu descobrir situações que teriam passado despercebidas sem sua utilização. Após analisar as entrevistas e os grupos de discussão, destacou-se a importante falta de reconhecimento que os participantes vivenciam, o que contribuiria, na opinião deles, para o surgimento da síndrome de *burnout*, bem como a falta de coesão coletiva.

## Abstract (es)

**Objetivo:**

el objetivo del presente estudio fue establecer los factores que inducen a los profesionales de enfermería a presentar *engagement* en su entorno laboral.

**Método:**

estudio cualitativo, usando la metodología comunicativa, entre profesionales de enfermería de centros públicos y privados de la ciudad de Huesca. Los discursos se recogieron mediante relatos comunicativos y grupos de discusión comunicativos.

**Resultado:**

la metodología usada ha permitido establecer un conjunto medidas de promoción del *engagement* en el entorno estudiado. Estas medidas se enmarcan en las tres categorías principales analizadas: las variables sistémicas o estructurales, las variables que se refieren al sujeto, y las que se refieren a las relaciones entre los sujetos.

**Conclusión:**

conocer cuál es la situación del *engagement* entre los profesionales, y las cuestiones que fomentan o dificultan su aparición, son fundamentales para poder establecer medidas que contribuyan a su desarrollo. El uso de las técnicas cualitativas ha permitido descubrir situaciones que hubieran pasado desapercibidas. Tras analizar las entrevistas y los grupos de discusión, ha quedado patente la importante falta de reconocimiento que experimentan los participantes, y que contribuiría, en su opinión, a la aparición del síndrome de *burnout*, así como la escasa cohesión como colectivo.

## Introduction

**Figure 1:**
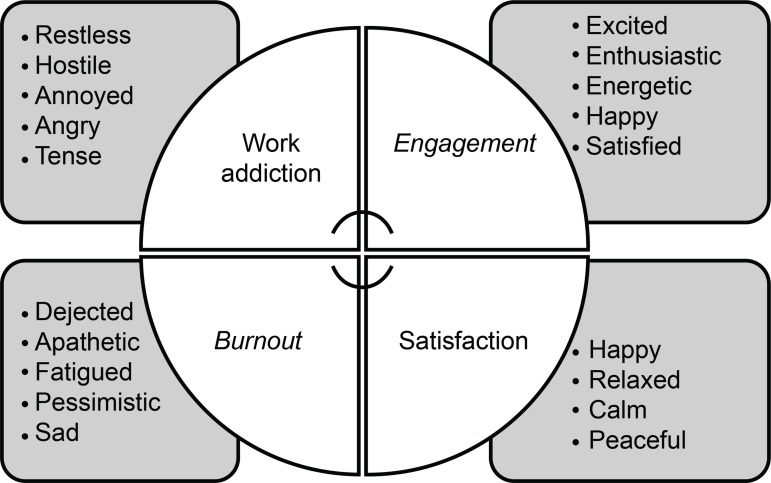
Two-dimensional model of subjective well-being at work

Source: adapted from Bakker & Oerlemans, 2012

Burnout syndrome should be understood as a form of psychosocial harassment at work, in which the workers are overwhelmed and powerless to deal with the problems generated by their work environment^(^ 1 ^)^; it was defined for the first time in 1974^(^ 2 ^)^.In opposition to this syndrome, the term engagement arises, reflecting workers who are engaged individuals, who take personal initiatives in their work, and generate their own feedback on performance^(^ 3 ^)^, while remaining aware that having high engagement does not mean ignoring the negative aspects of work and organizations ^(^ 4 ^)^.These terms also relate to the terms of work addiction and satisfaction, as shown in Figure 1 ^(^ 5 ^)^.

Burnout syndrome appears to a greater extent in those professions in whom continuous contact with people is required, so nursing has been one of the professions in which it has a higher prevalence: in Spain with 33%^(^ 6 ^)^, and internationally, reaching 55.3% in Brazil^(^ 7 ^)^, for hospital nurses in Colombia, 25.5%^(^ 8 ^)^, and 20% in Norwegian nurses specialized in obstetrics and gynecology^(^ 9 ^)^.

The high presence of burnout syndrome among health professionals in general, and nursing in particular, together with the interest of labor organizations to ensure professionals have a high level of engagement, caused the approach of the present investigation. In order to complement a previous study of quantitative type, the use of the qualitative communicative methodology was proposed, which can provide a novel and very interesting view of these concepts, as the type of study that addresses the prevention of burnout syndrome among nursing professionals follows cross-sectional^(^ 6 ^,^ 10 ^)^, or longitudinal, quantitative sampling^(^ 11 ^)^. Few studies use qualitative or mixed methods to address engagement among nursing professionals^(^ 12 ^)^, and none were found that used communicative methodology for this purpose.

The communicative methodology aims to describe reality, interpret and transform it, emphasizing how meanings are communicatively constructed from interactions between people on an equal level. The researchers will contribute the theoretical knowledge, and the participants, their personal experiences, establishing an equal dialogue at all times, where the contributions of the different participants are considered according to their validity, and not based on the position of power of those who are contributors^(^ 13 ^)^.

The communicative methodology not only aims to describe and explain, understand, and interpret reality with the aim of studying it, but also to study it in order to transform it, emphasizing how meanings are constructed communicatively through the interaction among people^(^ 14 ^)^.

The communicative methodology assumes a series of postulates, including: the universality of language and action, people as transforming social agents, communicative rationality, that is, that every person has the capacity for language and action, and common sense, understood as a subjective sense that depends on personal experiences that is formed within the cultural context. There is no interpretive hierarchy, since any assumption has the same strength, and both those being investigated and the researchers are on the same level (equal epistemological level)^(^ 14 ^)^.

The objective of this study was to establish the factors that induce nursing professionals to present engagement with their work environment, through a methodology rarely implemented on this subject: the qualitative communicative methodology.

## Method

The present study was conducted in all public and private health centers in the city of Huesca (Spain) that had nursing professionals within their workforce; the reference population, as of December of 2014, was527 nursing professionals. After conducting a quantitative study using standardized surveys, it was decided to conduct a qualitative study, using the communicative methodology, in order to perform a detailed discourse analysis and establish those elements that, in the opinion of the participants, favor the appearance of engagement. The participants in this qualitative study were those who indicated their desire to participate by completing the previous surveys, as well as professionals representing the group.

The statements were collected through communicative stories and communicative discussion groups. In this selection, the representativeness of each group was taken into account, as shown in Table 1.

**Table 1:** Characteristics of the participants in the study. Huesca, HU, Spain, 2016

Variables		Discussion groups (N=20)	Communicative stories (N=8)
Center type	Public	20	7
	Private	0	1
Type of contract	Permanent	8	7
	Temporary	12	1
Gender	Male	1	2
	Female	19	6
Specialty	Yes	2	1
	No	18	7
Work shift	Rotating	10	2
	Daytime	10	6

As for the communicative discussion groups, a maximum of 5 different nursing professionals were chosen to participate in a planned and designed conversation, with the purpose of confronting individual subjectivity with the group, to get their opinion about engagement in their work environment. A previous dialogue was held to share the objectives of the discussion group and explain to the participants the methodology being used.

Five discussion groups were held, in which 20 nursing professionals participated, whose characteristics are specified in Table 1.

Separating the supervisors or directors of the centers and units from the rest of the participants was taken into account when planning the groups, in order to avoid inhibition or coercion of the possible responses.

Regarding the communicative stories, an individual dialogue was held with representative professionals to reflect and interpret their daily lives in relation to professional engagement, with the purpose of detecting past, present, and future expectations.

A total of eight representative nursing professionals were interviewed within the city of Huesca, between April 12, 2016 and May 30, 2016. To complete the interviews, the interviewers went to the workplaces of the respondents, in order for them to feel comfortable in their environment, so that the discussion could be fluid. Those characteristics are specified in Table 1.

Scripts were prepared to guide the development of the conversation and to address the most important aspects related to engagement among the nursing professionals in the sample, taking into account the previous literature review.

These meetings were recorded and transcribed, and were held in a comfortable space for the respondents. Likewise, the objectives of the story were noted at all times, emphasizing that they are an active part of the research. In addition, informed consent was obtained to verify permission of the participants for the use of the data collected during the discussion groups.

In order to perform the content analysis, as a set of interpretative procedures of communicative products, in this case statements which came from previously registered communications^(^ 16 ^)^, the information was categorized into different levels of analysis, which were structured in a grid or table. This analysis was organized around the exclusionary and transformative components, characteristic of the communicative methodology, as well as around other components, such as transcription, coding, description, and interpretation of information^(^ 14 ^)^.

The exclusionary and transformative dimensions are^(^ 16 ^)^:

- Exclusionary dimensions: defined as barriers that people or groups encounter and that contribute to reproducing situations that favor the onset of burnout syndrome, and/or prevent the appearance of engagement.
- Transformative dimensions: defined according to the exclusionary dimensions, in that they are the opposite. These make it possible to overcome internal and external barriers, and prevent or hinder the appearance of burnout syndrome, favoring professional engagement.
- The variables analyzed were cataloged globally into three large groups, depending on whether the variables referred to the structural elements, the subject, or the relationships between the subjects. Within these groups, there were in turn specific subgroups of variables (Figure 2).
  **Figure 2:** Categories and subcategories of qualitative technique variables. Huesca, HU, Spain, 2016

Categories	Subcategories	
Systemic or structural	Organization	Organizational structure
		Schedules Teams/Loads Employment stability
	Physical environment	
	Bureaucratization / Computerization	
	Manager role	
	Dependent role	
	Professional skills	
Subject	Sociodemographic variables	
	Professional experience	
	Personality	
	Education	
	Implication	
Relationships between subjects	Among professionals	
	With the patients	
	With the relatives	
	Social assessment

The variables were assigned codes, creating an analysis matrix according to the defined categories and subcategories (Table 2). This allowed assigning said codes to the different analysis units found in the participants’ statements.

**Table 2:** Exclusionary and transformative dimensions. Huesca, HU, Spain, 2016

Variables			Exclusionary Dimensions	Transformative Dimensions
Systemic / Structural	Organization	Organizational structure	1	10
		Schedules	2	11
		Teams	3	12
		Employment stability	4	13
	Environment		5	14
	Bureaucratization		6	15
	Manager role		7	16
	Dependent role		8	17
	Professional skills		9	18
Subject	Personal variables		19	28
	Professional experience		20	29
	Personality		21	30
	Education		22	31
	Implication		23	32
Interrelations ships	Among professionals		24	33
	With patients		25	34
	With relatives		26	35
	Social assessment		27	36

After coding the information, the different analysis units coded according to the analysis grid were grouped, to proceed at a later point to their description and interpretation.

## Results

The results obtained in this study will expose those expressions or statements valued as transformers, that is, that promote the appearance of engagement in the work environment, and in turn, can be assessed as proposals for improvement for workplaces, where they can be implemented to combat the onset of burnout syndrome.

Analyzing the discourse of the participants in search of those elements considered transformative has allowed the establishment of a set of measures to promote engagement in the studied environment. Next, these measures are exposed according to the three main categories analyzed: systemic or structural variables, variables that refer to the subject, and those that refer to the relationships between the subjects. Within these main categories, the most specific ones will be developed, as specified above.

Regarding the structural category, the first measure proposed is the existence of agreements at the national level, since it would avoid the changes suffered by public health guidelines in Spain: *And at the political level, make a good pact for health where certain things that are the pillars support it, because they were maintained over time regardless of the administration* (EEDH*: 112, 10).

On more specific levels, a significant number of participants suggest that the occurrence of burnout syndrome could be prevented by performing work by objectives: *If we encourage teamwork, we would all know that everyone has a part in that job. And that you also have to have time to do it* (EESMB: 110, 10).

Some of the participants propose that, from nursing collegial organizations, professional unity should be promoted by carrying out group activities. Collegiate organizations should, in their opinion, promote greater visibility of the profession: *I believe that we have to move society to understand[what] a nurse is,and really explain what nursing does, we fail nursing on this, so the population really doesn’t know everything we do* (EEDH: 54, 18).

As for work schedules and shifts, they propose that to promote engagement in the work environment, a time control should be established for all interdisciplinary team professionals, which would foster feelings of equality.

In their opinion, not only should the ratio of patients per nurse be assessed, but also the characteristics of these patients, since there are care units with low patient ratios per nurse, in which the care they require is very extensive and represents a significant workload, which favors the onset of burnout syndrome.

Following with measures at a structural or organizational level, some of the participants propose the creation of meeting points: *I believe that there is no place where you can complain, and that the rest of the people can find out* (GD5F: 1021, 10).

Most of the participants propose that, for the organization of workload, the opinion of those involved should be taken into account, which could arise from the meeting points mentioned above.

Most participants propose the professionalization of health management as a tool for the promotion of engagement. Thus, this professionalization of management would improve the link between professionals, by promoting feelings of respect and union: *A change in these organizations that represent us, or for example in the nursing departments, that is to say, more professionalized nursing departments would unite us more as a profession* (EESCM: 189, 10).

Referring more specifically to leadership, and related to the proposals above, there is a broad consensus among the participants regarding the proposal of leaders who exercise their roles with transparency, and foster feelings of belonging to the company where they work.

When referring to the relationship between leadership and recognition as tools for the promotion of engagement, in the opinion of most participants, recognition by superiors should be improved: *Improve leadership capabilities* (EESTM: 164, 16).

Other measures proposed at the structural or organizational level could be the existence of support professionals such as psychologists, who could assist the different nursing professionals in preventing burnout syndrome, and promote engagement in the work environment: *Let’s see, I also believe that if one day you have too much, then you have a psychologist or you have something to say ... well look I’m upset about this and I need help* (GD2A: 387, 10).

With the purpose of improving family reconciliation, some of the professionals interviewed propose the existence of childcare centers in the facilities, a measure which would enable workers to see a favoring of the reconciliation between their private life and their work life, which could favor the development of engagement.

Continuing with the structural or organizational proposals, we found those referring to improved working conditions. Most of the participants considered that improvement in employment contracts and in labor stability within the public employment system would promote the appearance of engagement, through improvements in the system for the provision of hospital posts, and a greater number of job offers in public employment to reduce temporary work. In this way, they stated that it would promote engagement: *Well, publishing the lists of fixed posts* (GD2B: 335, 13).

Most of the participants proposed that computer programs that are implemented should be simpler, intuitive, and the same for the whole health center, thus avoiding the variability between the services: *And make it practical and ... intuitive* (EESMA: 335, 15). Likewise, they propose that there be a period of sufficient and adequate adaptation and training, prior to the computerization of services, and even: *That the program can’t be done by a computer scientist without a nurse right beside him* (EESMA: 325, 31).

Finally, within the proposals at the organizational level, some of the participants believe that the promotion of research within the work environment would favor the appearance of engagement: *(…) Promote research at the nursing level. It’s very important. Because, although we think it’s not true, having papers published in journals, in whatever medium, is important* (EESTM: 246, 31).

Within the organizational interventions, those aimed at increasing the level of knowledge of individuals about burnout syndrome and engagement in the work environment can also be placed, and these proposals can also be placed within individual interventions. Some of the participants believed that knowing these constructs could help in their identification.

When referring to the category of the subject, some of the participants thought that the development of engagement could be encouraged by improving knowledge on workers’ health management: *In the management career, everyone passes like it’s nothing, and I think we should be taught more (...), if you knew more about management, you would manage even your work, and your cure, right?* (EESCM: 74, 31).

Another line of training proposed by the participants would be aimed at training in leadership skills, and in promoting research.: *(…) Promote research at the nursing level. It’s very important. Because, although we think it’s not true, having papers published in journals, in whatever medium, is important (…)* (EESTM: 246, 31).

One of the proposals focuses on receiving training related to the development of optimism and humor in the work environment: *I think that is positive, I mean, the teams ..., seeing things in a positive way and seeing things with humor is important because if not ..., you would go crazy* (GD4C: 443, 30).

On the other hand, the participants proposed training to learn to separate or complement work life and family life could help prevent the onset of burnout syndrome.

Other lines of training proposed to promote the appearance of engagement would be training in communication skills, social skills, interpersonal relationships, and teamwork: *Learn social skills not as protection, but learn social skills to, say, be able to provide greater comfort to family members and patients, while at the same time avoid suffering from burnout…* (EEDH: 38, 31).

Finally, the participants proposed training in how to proceed during work shift changes, what to say, how to say it, and where to do it, in order to learn how to save time, protect patients’ personal data, and improve continuity of care: *Well, a protocol is necessary, something standardized. It would probably be good.* (EESMA: 365, 33).

As for relationships with other professionals, there is a broad consensus that the promotion of teamwork would promote the existence of engagement. In their opinion, this could be done through interdisciplinary meetings and lines of communication between the different professionals: *Establish lines of communication between different professionals, this would increase professional commitment* (EEAP: 163, 33).

In order to improve teamwork with the different professionals, and not perform functions that are not typical of nursing professionals, participants thought that a measure that would favor engagement would be the training them to know their own and other people’s functions: *Well, of course, (...) the competences of each one, as they are multidisciplinary teams that coexist with different professions, because there are a number of limits that do not have to be frontiers, but that complement each other* (EEMH: 40, 18).

Most of the participants propose that the nursing profession be publicized because, in their opinion, it would increase the social recognition of the profession: *Tell the population what we do. That we not only give the pills and give the injections. And that would improve people’s knowledge of the profession. (…) And that improves the fact of how others see us* (EESTM: 176, 36).

## Discussion

The welfare of workers within the work environment is an important part of the efforts of companies and organizations, this is especially relevant among companies dedicated to health care. Knowing the variables associated with engagement and burnout syndrome, within a specific health environment, will provide the relevant tools to implement plans and strategies that foster engagement, so as to promote worker welfare, improve the quality of the services and care offered, and reduce patient safety.

Most of the proposals put forward by the participants coincided with previous proposals exposed by other authors, such as the reflection on the size of the centers and the cities of work; in their opinion, this represents an important influence on the burnout syndrome^(^ 17 ^)^. Small centers are more accessible, comfortable, and less stressful, which could favor the development of engagement. On the other hand, some participants suggested that the environment in which the study was conducted greatly encouraged post-university training, and that this fact could also favor the development of engagement.

Most participants state that the gender of professionals influences the relationships established between them, with other professionals, and with families and patients. In addition, participants emphasize the fact that the majority of nursing professionals are women, since until recently work was considered secondary for women, while this did not occur among the group of men. In the opinion of the participants, this would also be related to marital status and family-work conciliation, since, in their opinion, these facets are difficult to separate, and the conciliation between the two is complex, which can favor the development of burnout syndrome. Previous studies conclude that femalesappearmore likely to suffer from burnout syndrome, since they have higher percentages of high levels of exhaustion, cynicism, and less efficacy than do males, but this relationship isunclear. It could be explained by considering thatthe experience of stress seems to be linked to gender roles^(^ 18 ^)^.

Participants associate job instability with greater insecurity, which makes reconciliation difficult, decreases the quality of care, and hinders continuous training, making it difficult to develop engagement. In the opinion of the participants, labor instability is fostered by health organizations, in order to give workers less rights than those who have a fixed contract, although in the previous bibliography emotional fatigue is associated with having a fixed post^(^ 19 ^)^.

In the opinion of the participants, having professional leaders who combine transparency in management and professionalism would increase the link between professionals and the organization, and could favor the development of engagement. Likewise, in their opinion, if organizations fostered feelings of belonging, this would lead to a higher level of commitment among professionals, and a lower level of burnout syndrome among them.These data are corroborated with previous studies where it is concluded that nursing managers are key to the promotion of professional commitment or engagement, promoting the improvement of leadership behaviors, and an environment of optimism and self-efficacy that increases work commitment^(^ 20 ^)^.

Health professionals feel that the organizations they work for treat them unfairly, so developing models that improve these feelings of justice would directly improve the engagement of individuals with the organization. These results are similar to those in other studies ^(^ 21 ^)^, where it is observed that when organizations behave with greater justice, nurses have less burnout syndrome.Measures aimed at developing this sense of justice could be the implementation of transformational leadership models^(^ 22 ^)^, or the aim towards the objectives, which has been found to be related to occupational well-being^(^ 23 ^)^.

The relationship between workload and the appearance of burnout syndrome is referred to in the interviews and discussion groups conducted. In them, the participants indicate that the lack of personnel and material resources increases the level of stress, and promotes the appearance of burnout syndrome, in the same way that other authors have associated it previously^(^ 24 ^)^.

Participants state that the lack of training at university in time control skills, emotional management, communication skills, and social skills contributes to the appearance of burnout syndrome. Previous studies conclude that lack of control and overload are estimated to be the main sources of stress^(^ 25 ^)^. Training in these areas would enable agreater capacity to manage complex situations of daily practice, offer greater comfort to users, and increase trust among professionals.

Participants suggested that there is a lack of recognition ofnursing professionals,by managers and society in general, which prevents the development of engagement. This lack of recognition refers to the reward, understood as an institutional, social, or financial reward, and it is observed that, if this is insufficient, there is a greater risk of suffering from burnout syndrome^(^ 26 ^-^ 27 ^)^.

On numerous occasions, participants expressed the importance that their own evolution had onthe nursing profession, sometimes considering it as unprofessional, or very dependent on other professions with which they work in a coordinated manner. This, together with the important recognition of the female profile of the profession, is considered a key factor that can facilitate the onset of burnout syndrome, since gender is considered by the participants as a determinant in the relationships between professionals.

Currently, although women represent 84.3% of the registered nursing professionals, only 43.48% of the presidents of the provincial nursing colleges are women. Although female representation is the majority in nursing, these professionals occupy positions of lower responsibility in their collegiate organizations.These facts contribute to perpetuating situations of inequality, which must be reversed by promoting, through training, actions that promote the incorporation of female nurses to positions of responsibility^(^ 28 ^)^.

The confrontation in the definitions of nursing as a vocation or as a profession is, therefore, a continuing fact that impacts the current reality, althoughcontradictory opinions were obtained among the participants of the present study. This aspect should be studied more deeply, and the communicative methodology could provide an innovative viewin terms of its approach.

Regarding the relationships between professionals, the importance of having healthy interprofessional relationships in order to achieve engagement in the workplace is clear^(^ 29 ^)^.

If reference wasmade to the relationships with the patients and their characteristics, the participants stated that continuous contact with disease and suffering favors the onset of burnout syndrome.Similar conclusions wereobtained previously by other authors, where it wasobserved that continuous care forpatients who have high levels of suffering causes negative emotional reactions among health professionals^(^ 30 ^)^.

Regarding the relationships with the relatives of the patients under the care of the nursing professionals, they stated that these relationships werecomplex, with an increase in the demands and those that hinder the development of engagement in the work environment, a fact that is seen also hindered by the increase in aggressions and verbal violence towards health professionals. Other authors arrive atthese same conclusions, which relate the existence of aggresion with the appearance of burnout syndrome^(^ 31 ^)^,exposure to verbal abuse with high levels of emotional fatigue, depersonalization, and intention to leave work^(^ 32 ^)^.

Improving and implementing job engagement in nursing professionals prevents the risk of suffering job stress and burnout, and consequently leads toa higher quality of care for users of their services. Knowing the engagement situation among professionals, and the issues that, in the opinion of those involved, encourage or hinder their appearance, isessential to establish measures that contribute to their development.

The use of qualitative techniques has allowed the discovery of situations that would have gone unnoticed otherwise, identifying a series of risk factors that were not easily identifiable through quantitative techniques.

The main limitation of the study is that it was developed in a specific area of northeastern Spain, so the results may not be representative of nursing professionals in other territories. Despite this, the study was conducted in health centers with a capacity of less than 500 beds, and these centers represent 90% of Spanish health centers^(^ 33 ^)^. On the other hand, there is the possibility that some of the participants have modified their responses based on what they thought was expected of them (social desirability bias).

## Conclusion

After analyzing the interviews and the discussion groups, it can be concluded that there are different proposals that act as transformative elements, hindering the appearance of burnout syndrome, and favoring the appearance of engagement. The main transformative proposals are:

- decrease the size of work centers,
- favor the continuous training of nursing professionals, emphasizing training in communication skills, emotional management, time control, social skills, and knowledge of the concepts of engagement and burnout,
- improve the reconciliation of family life and working life,
- improve job stability,
- improve leadership among nursing professionals, and foster feelings of belonging to the organization,
- improve feelings of justice in the workplace,
- fight against the lack of personnel and material resources in the work environment of nursing professionals,
- improve recognition and reward of nursing professionals, both institutionally, socially, and in the family,
- promote gender equality in the organizational representation of nursing professionals, breaking the “glass ceiling” currently in existence,
- favor healthy and egalitarian interprofessional relationships,
- control and reduce verbal and physical violence in the healthcare environment.

The use of the communicative methodology will allow the design of prevention strategies that work to transform areas that favor the appearance of engagement.One of the preventive strategies may be to hold meetings similar to the discussion groups, and convert the dialogic methodology into a prevention proposal.

## Footnotes

* Acronyms have been used to ensure the anonymity of participants in interviews and discussion groups conducted. EEDH: Male Management Nurse Interview; EEAPM: Female Nurse Primary Care Interview; EEMH: Mutual Male-Male Interview; EEPH: Male Nurse President Interview; EESMA: Female Nurse Union Interview A; EESMB: Female Nurse Union Interview B; EESTNM: Female Nurse Supervisor Evening Nights Interview; EESCM: Female Nurse Supervisor Contracted Interview; GD1A, GD1B, GD1C, GD1C: Discussion Group 1 individual A, B, C or D, respectively; GD2A, GD2B, GD2C, GD2C: Discussion Group 2 individual A, B, C or D, respectively; GD3A, GD3B, GD3C, GD3C: Discussion Group 3 individual A, B, C or D, respectively; GD4A, GD4B, GD4C: Discussion Group 4 individual A, B or C, respectively; GD5A, GD5B, GD5C, GD5C, GD5F: Discussion Group 5 individual A, B, C, D or F, respectively.
